# On Some Minor Anal and Rectal Ailments—II

**Published:** 1895-03-02

**Authors:** W. McAdam Eccles

**Affiliations:** Assistant Surgeon West London Hospital and City of London Truss Society, Surgeon St. Marylebone General Dispensary, and Senior Assistant Demonstrator of Anatomy St. Bartholomew's Hospital


### Medical Progress and Hospital Clinics.

[*The Editor will be glad to receive offers of co-operation and contributions from members of the profession. All letter should be addressed to* The Editor, The Lodge, Porchester Square, London, W.]

### ON SOME MINOR ANAL AND RECTAL AILMENTS.—II.

By W. McAdam Eccles, M.B., M.S.Lond., F.R.C.S. Eng., Assistant Surgeon West London Hospital and City of London Truss Society, Surgeon St. Marylebone General Dispensary, and Senior Assistant Demonstrator of Anatomy St. Bartholomew's Hospital.

**Pruritus ani** is another condition which may render a person's life absolutely miserable, and yet there may be little to show as to its cause. It must be remembered that the affection is really a symptom, and as such cannot be relieved unless the source of the trouble is attacked. While admitting that in certain cases it may undoubtedly be a pure neurosis, yet I am sure the majority of instances have a definite lesion as their basis. Dirt of various kinds is a factor in its production, but as it is very frequent among the most cleanly it cannot be said to play a universal part. In my experience, those of a gouty diathesis are infinitely more prone to this distressing complaint than others.

Eczema in the region of the anus is probably the most usual local cause, and this, again, is frequently associated with gout. This eczema may, however, be the result rather than the cause of the itching in a certain proportion of cases; but when the eczema is acute, and there is a red, angry surface exuding a gummy discharge, it is probably primary, often associated with some smarting pain in addition to the irritation. Though bothered during the day, the patient's misery becomes greatly increased at night, presumably because his mind is freer to dwell upon his affection. Sleep is disturbed, and the general health suffers much. These acute cases are usually the more easy of the various forms to cure. From the outset it is essential that soothing applications be used. I have found dry applications answer better than lotions, and I think the patient finds these applications much more simple. Very fine boracic acid powder, with one-half the quantity of zinc oxide, is excellent. Great attention must, however, be paid to the details of its application. The plan I usually follow is that first the parts are to be cleansed with a very weak oatmeal gruel, then dried with a soft handkerchief, and, having freely dusted a piece of lint of a suitable size on the smoother surface with the above powder, it is applied to the raw surface, and kept in place with the firm pressure of a pad of antiseptic absorbent wool and a T-bandage. This should be changed at first about every four hours, but usually a case markedly yields to such treatment in three days.

Chronic eczema is much more difficult of cure, and so is its ally, sub-acute eczema. Here the area around the anus will be found dry and cracked, and often of a whitish appearance, the natural pigment having disappeared. I am quite certain that tar preparations are the most suitable in these cases. Van Buren suggested an ingenious method for their application in the form of a pad of carefully prepared oakum kept in contact with the parts with some degree of pressure. It is very necessary that the affected regions be kept from coming in contact with one another, and this such an application will successfully achieve. Painting with boroglyceride I have seen great benefit from in one or two cases which did not yield to the tar treatment. In very obstinate cases removal of the skin over the greater part of the affected area may be advisable. Eczema marginatum, caused by the parasite trichophyton, may involve the anus. This is very tedious in its cure, but an ointment of a 5 per cent. strength of pyrogallic acid is most likely to be efficacious.

Thread Worms (oxyuris vermicularis) are by no means infrequently a cause of intolerable itching about the anus. These worms do not have their habitat in the rectum as a rule, being chiefly found in the lower part of the small intestine and the cæcum; the females, however, descend into the rectum to deposit their eggs. It will thus be seen that there must necessarily be difficulty in removing this source of the irritation. I believe that free purging answers much better than injections into the rectum, though these may be combined with the former.

Other parasites, as pediculi and scabies, may possibly be causes of pruritus. Internal hæmorrhoids, it must be borne in mind, do occasionally act in the production of the symptom.

As to the treatment of cases where no local affection can be demonstrated, it may be said that probably but little good will be obtained except with a long, steady, persevering course of treatment. Chloroform ointment will relieve one case, and cocaine another, but I always try the effect of keeping the bowels acting freely, bathing the parts with very hot water just before retiring for the night, and the application of an ointment of oleate of mercury containing a smal quantity of morphia. If this prove unsuccessful after a thorough trial, I advise dilatation of the sphincter and the removal of a portion of the skin around the margin of the anus.

**Anal Condylomata.**—These oval, raised, whitish, symmetrical, sodden patches of hypertrophied epithelium are extremely frequent during the secondary stage of acquired or inherited syphilis, especially where the parts are not thoroughly cleansed. It must, however, be remembered that condylomata are not always the outcome of syphilis, any more than vulval warts are uniformly indicative of gonorrhœa. There is a very fœtid secretion from these patches which is highly contagious. They sometimes run on into troublesome ulceration. Besides constitutional treatment for syphilis, I have found the plan of procuring great cleanliness answers perfectly, but a local application, after washing and drying the area, of a powder containing calomel, iodoform, and zinc oxide or starch is useful. Papillomata about the anus seems to be caused in much the same way as condylomata. The actual cautery should be employed if they are intractable.

**Vascular Patches of the Rectal Mucous Membrane.**—Patients have several times come under my notice who, after every motion, lose a certain amount of blood, more if there is constipation, less if the stool is soft. On careful examination no ordinary venous pile can be found, but one, two, or more patches from which blood will ooze under the very slightest provocation. In some cases of intense anæmia the cause of the bloodlessness has been traced back to the loss occasioned by such a condition. A view obtained by a rectal speculum is advantageous.

What should be the treatment of these troublesome cases? Firstly, the motions must be kept soft; and secondly, an astringent ointment to be introduced within the anus before and after an evacuation should be recommended. Ten grains of the sulphate of iron to an ounce of vaseline is very efficacious. If the condition does not rapidly yield to this treatment, it is well to dilate the anus and apply to the area of the patches themselves some strong nitric acid, or the actual cautery.

**Superficial Ulceration of the Rectal Mucous Membrane.**—There appear to be two chief causes of this rectal disease, and although the local condition may seem slight, yet, as it is dependent either upon tubercle or nephritis, it is really a serious matter. The patient will be troubled with a blood-stained muco-purulent discharge, accompanied by pain if near the anus. Nothing appears of much service in relieving the complaint, and it is an unfortunate fact that the disease is not uncommonly a fatal one.

In the palliative treatment of these distressing cases two points are to be borne in mind—(1) that there should be as little irritating material brought to the ulcerated part from above as possible, and (2) that the harmful discharges from the inflamed area be got rid of as promptly as circumstances will allow. To effect the former, an exclusively milk diet may be tried; to secure the latter, frequent washing out of the bowel—boracic acid lotion is needed—performed with great care and gentleness. A starch and opium enema after the last washing for the day will be greatly appreciated by the patient. It is advisable to keep patients affected with rectal ulceration in bed for a time while treatment is being carried out. I am by no means sure that any ointment introduced into the bowel is of much service.

Constitutional treatment should, of course, be combined with the local treatment, and if there be any suspicion of a syphilitic taint appropriate remedies are to be persevered in.

In some cases benefit arises from a division of the sphincter, and drainage of the lower end of the bowel, but there is some likelihood of the wound refusing to heal perfectly.

Occasionally when the ulceration is extensive, and the patient is getting exhausted by pain and discharge, an inguinal colotomy may give relief, affording as it does the prevention of the passage of fœcal material over the ulceration, and the opportunity of more thoroughly and frequently washing out the discharges

